# Quantitative MRI Demonstrates Abnormality of the Fornix and Cingulum in Multiple Sclerosis

**DOI:** 10.1155/2013/838719

**Published:** 2013-02-16

**Authors:** Stephanie B. Syc, Daniel M. Harrison, Shiv Saidha, Michaela Seigo, Peter A. Calabresi, Daniel S. Reich

**Affiliations:** ^1^Department of Neurology, Johns Hopkins University, School of Medicine, Baltimore, MD 21287, USA; ^2^Translational Neuroradiology Unit, National Institute of Neurological Disorders and Stroke, National Institutes of Health, 10 Center Drive, MSC 14001, Building 10/5C103, Bethesda, MD 20892, USA

## Abstract

*Objective*. To characterize MR signal changes associated with tissue damage in the fornix and cingulum in multiple sclerosis (MS) using quantitative MRI measures and to determine associations with cognitive dysfunction. *Background*. The fornix and cingulum are white-matter bundles that carry information related to cognition. While cognitive dysfunction is reported in 40–60% of MS patients, the neuroanatomical correlates of cognitive impairment remain incompletely understood. *Methods*. The cingulum, pillars of the fornix, and corticospinal tract were segmented by fiber tracking via diffusion tensor imaging. Average tract-specific fractional anisotropy (FA), mean diffusivity (MD), and magnetization transfer ratio (MTR) were compared in MS cases and healthy volunteers. Associations with clinical measures and neuropsychological tests were derived by multivariate linear regression. *Results*. Fornix FA (*P* = 0.004) and MTR (*P* = 0.005) were decreased, and fornix MD (*P* < 0.001) and cingulum MD (*P* < 0.001) increased, in MS cases (*n* = 101) relative to healthy volunteers (*n* = 16) after adjustment for age and sex. Lower fornix FA and MTR, and higher fornix MD and *λ*_||_, were correlated with lower PASAT-3 scores, but not with slower 25FTW times. Lower PASAT-3 scores were associated with lower cingulum FA and higher MD and *λ*_⊥_. *Conclusions*. Cognitive dysfunction in MS may involve damage to a widespread network of brain structures, including white-matter pathways within the limbic system.

## 1. Introduction 

Cognitive dysfunction is reported in 40–60% of MS cases, can be present at both early and late stages of the disease, and can occur even in the presence of low overall physical disability [1–3]. MS-related cognitive dysfunction is most commonly characterized by relative decline of neuropsychological tasks assessing processing speed, short-term memory, attention, visuospatial abilities, and executive functioning [4, 5]. These deficits can negatively impact quality of life, as people with cognitive impairments are less likely to be employed, run a household, or participate in social and vocational activities [6]. 

Cognitive deficits may be subtle and unreliably detected in clinical evaluation of MS [7], and their neuroanatomic basis is incompletely understood. Many studies have used MRI to investigate the relationship between structural damage and cognitive impairment. Cognitive impairment has been associated with frontal and temporal lobe T2-weighted lesion load, T1 “black holes” (indicative of more severe tissue damage within lesions), and brain atrophy [8–13]. The corpus callosum, the major white-matter structure connecting the right and left hemispheres, is affected in MS, and callosal damage has resulted in impairment in complex tasks and cognition [14–16]. Studies have also shown that atrophy of gray-matter structures, including the cerebral cortex, contributes to disability and neuropsychological deficits [17–22]. In particular, atrophy of some cortical regions, especially the hippocampus [23], has been associated with worse performance in cognitive tasks in early stages of the disease, whereas other MRI measures (such as white-matter volume) do not differ between cognitively impaired and cognitively preserved cases [3]. Postmortem analysis has also demonstrated demyelinating lesions within the hippocampus [24–26].

The central role of the hippocampus in complex cognitive functions raises the possibility that the major hippocampal afferent and efferent pathways may also be involved. We, therefore, undertook a study of the fornix and cingulum, white-matter bundles within the limbic system that are associated with memory functions [27–29]. The fornix forms one of the major hippocampal efferent pathways, connecting the hippocampus with the mammillary bodies and septal nuclei. The cingulum bundle connects the limbic system to the cerebral cortex, including the cingulate gyrus. The anterior cingulate gyrus is known to moderate executive control of attention, whereas the posterior cingulate gyrus is important for orientation and spatial attention [30–32].

Quantitative MRI indices derived from diffusion tensor imaging (DTI) and magnetization-transfer-weighted imaging have been used to assess microstructural damage to white-matter tracts and its relationship to disability in a variety of functional systems [16, 33–35]. In this retrospective study, therefore, we investigated whether quantitative MRI measurements in the fornix and cingulum differ between MS cases and healthy volunteers and evaluated the association of these measures with cognitive disability. 

## 2. Methods 

### 2.1. Subjects

The study protocol was reviewed and approved by the Johns Hopkins Institutional Review Board. Informed consent was obtained from all study participants before study procedures were conducted. Study participants were recruited from the Johns Hopkins MS Center, and healthy volunteers were recruited from the Johns Hopkins community. For the MS cases, a convenience sample was used. Inclusion criteria included a confirmed diagnosis of MS, made by the treating neurologist, and the ability to undergo an MRI exam. All MS clinical subtypes were included. Individuals with neurological conditions other than MS were not studied, nor were participants whose scans were conducted within six weeks of corticosteroid treatment. Cognitive testing and neurological examination were completed within six weeks of the MRI scan. 

### 2.2. MRI Acquisition

MRI scans were acquired on a 3-tesla scanner (Philips Medical Systems), and sequence parameters and image reconstruction have been described in detail elsewhere [16]. Data were collected over a 27-month period, from March 2008 to May 2010. From the DTI data, we derived maps of FA, MD, parallel diffusivity (*λ*_||_), and perpendicular diffusivity (*λ*_⊥_). From the magnetization-transfer data, we calculated the MTR, which is the fractional signal decrease after application of an off-resonance radiofrequency pulse. 

### 2.3. Fiber Tractography

The cingulum and the body and pillars of the fornix were identified by the Fiber Assignment by Continuous Tracking method implemented through DtiStudio version 2.40 (http://www.mristudio.org) [36]. An FA threshold of 0.13 was used as the criterion for starting and stopping tracking. This value was much lower than average intralesional FA values, but it is possible that the presence of lesions resulted in exclusion of some portions of the cingulum and fornix in some cases (although visual inspection showed that this was not a substantial problem in our data). A turning angle of 40° was used as a stopping criterion to terminate individual streamlines. These parameters are similar to those used in other studies of our cohort [16] and allowed reconstruction of tracts in all study participants.

**Figure 1:**
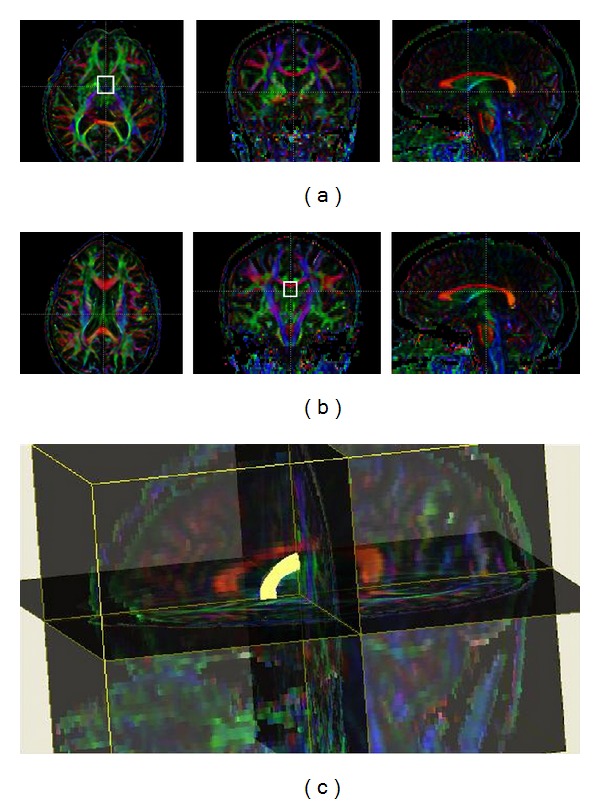
Tractography of the fornix. Regions of interest used for tractography of the fornix were drawn one axial slice above the anterior commissure (a) and at the hippocampal commissure on coronal reconstructions of the DTI color maps (b). Streamlines were required to pass through both regions of interest and were terminated at those points. Three-dimensional reconstruction of the fornix in an MS case (c).

**Figure 2:**
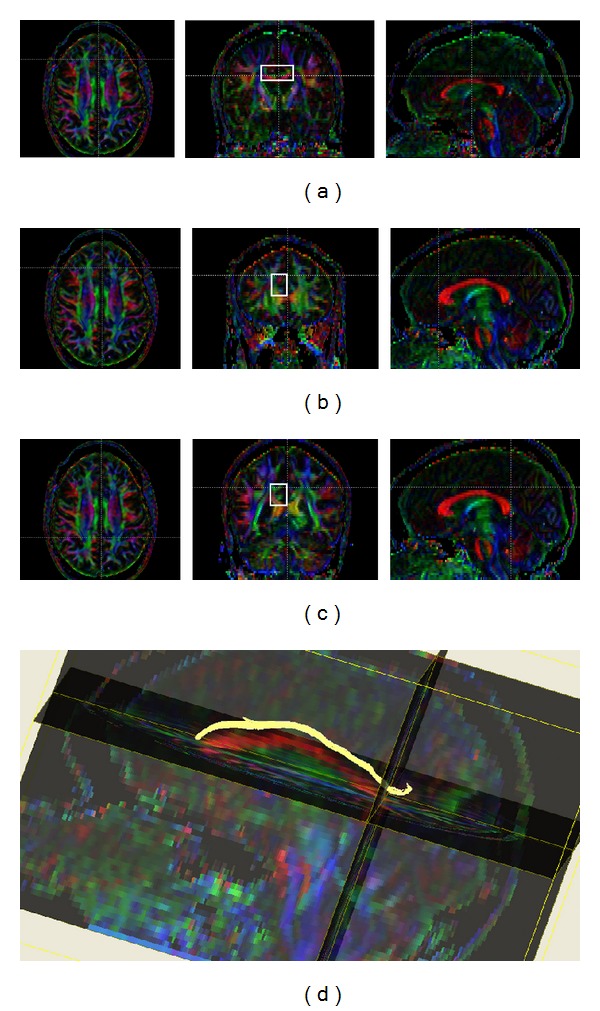
Tractography of the cingulum. Regions of interest were drawn on coronal sections. The first region was drawn on a coronal slice through the body of the corpus callosum, where cingulum fibers have an anteroposterior orientation (a). The second (b) and third (c) regions were drawn surrounding the area directly rostral to the corpus callosum at its anterior and posterior tips. The left and right cingula reconstructions were completed individually. Streamlines that passed through all three regions of interest were included in the study analysis and were terminated at the anterior and posterior regions. Three-dimensional reconstruction of the left cingulum in an MS case (d).

Regions of interest for fornix reconstruction were placed one axial slice above the anterior commissure and at the hippocampal commissure on coronal reformations of the DTI color maps. For the cingulum, regions of interest were placed rostral to the corpus callosum on a coronal slice through the midbody and also above the anterior and posterior tips of the corpus callosum. Fibers that passed through all regions of interest were analyzed (Figures 1 and 2). Right and left cingula were reconstructed in separate steps. After reconstruction, MRI indices derived from the two cingula were averaged across the right and left sides. 

Corticospinal tract MRI indices were measured in order to evaluate nonspecific associations with cognitive dysfunction (i.e., association with overall disease burden), since the corticospinal tract is primarily involved in movement. These data were derived via a previously published atlas-based method, based on tract probability mapping, in all scans [37]. 

### 2.4. Disability Measures and Cognitive Testing

Expanded Disability Status Scale (EDSS) evaluations were conducted on all MS cases by a Neurostatus-certified EDSS rater [38]. Timed 25-Foot Walk (25FTW), 9-Hole Peg Test (9-HPT), and Paced Auditory Serial Addition Task-3 (PASAT-3) were completed in the MS cases as part of the Multiple Sclerosis Functional Composite (MSFC) assessment. Two runs of the MSFC assessment were performed to mitigate learning effects. These tasks were administered, and *z*-scores were calculated, following the protocol described in the National Multiple Sclerosis Society's MSFC Administration and Scoring Manual. 25FTW and 9-HPT data were available for 101 MS cases and PASAT-3 for 100 cases (1 participant refused to complete the task). 25FTW data were averaged over two trials, and 9-HPT was averaged over the dominant and nondominant hands. 

### 2.5. Statistical Analysis

Values are reported as mean ± standard deviation except where otherwise indicated. The Wilcoxon rank-sum test was used to compare MRI indices between MS cases and healthy volunteers. Associations with MSFC components, disease duration, and EDSS score were derived by multivariate linear regression, adjusting for age and sex as additive factors, using Stata (version 11). We report partial Pearson's correlation coefficients (*ρ*) and their associated *P*-values without adjustment for multiple comparisons. Pearson's correlation coefficients (*r*) are reported for the association between MRI measures and age. 

## 3. Results

This study population consisted of 101 MS cases and 16 healthy volunteers (Table 1). The majority of cases had relapsing-remitting MS (RRMS; *n* = 64, 63%). The secondary progressive MS (SPMS) and primary progressive MS (PPMS) cohorts were significantly older (SPMS: 55 ± 8 years, PPMS: 56 ± 7) than the RRMS cohort (39 ± 11). The average age of the healthy volunteer cohort (40 ± 9) was not different from that of the MS cohort (*P* = 0.26).

**Figure 3:**
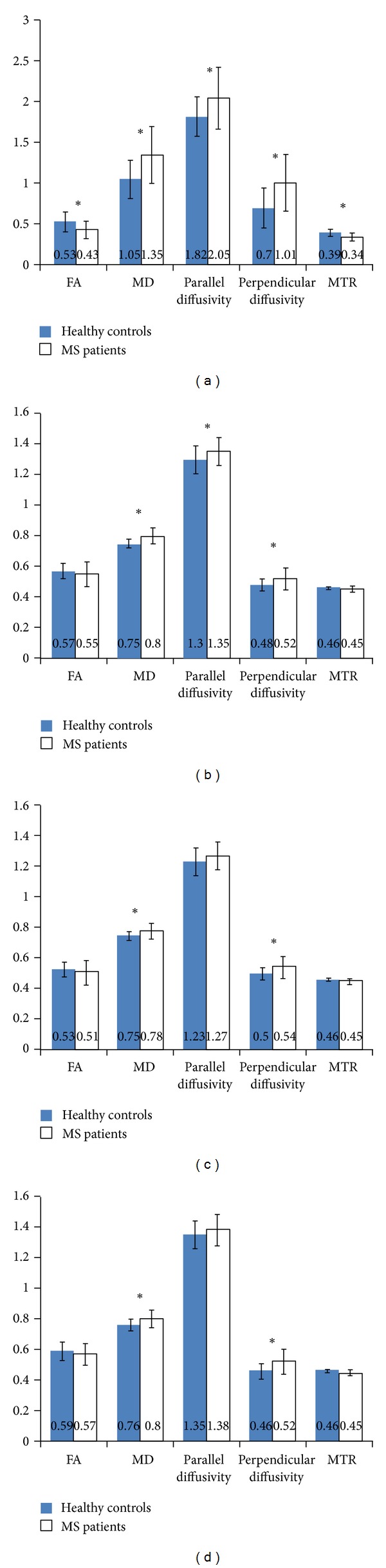
Mean MRI indices in MS cases and healthy volunteers across the fornix (a) and averaged cingula (b), left cingulum (c) and right cingulum (d). *Indicate significant differences (*P* ≤ 0.05) between the two groups. FA: fractional anisotropy, MD: mean diffusivity, and MTR: magnetization transfer ratio. Error bars represent the standard deviations of the measures.

Fornix and cingulum MRI indices were abnormal in MS cases relative to healthy volunteers. Fornix FA (*P* = 0.004) was decreased by 19%, and MTR (*P* = 0.005) by 13%, in MS cases. Fornix MD (*P* < 0.001; 29%), *λ*_||_ (*P* = 0.008, 13%), and *λ*_⊥_ (*P* < 0.001; 44%) were increased in MS cases. Averaged across the right and left sides, cingulum MD (*P* < 0.001, 7%), *λ*_||_ (*P* = 0.02, 4%), and *λ*_⊥_ (*P* = 0.02, 8%) were increased in MS cases. Separately, MD and *λ*_⊥_ were increased on both the right (MD: *P* = 0.009, 4%; *λ*_⊥_: *P* = 0.02, 8%) and left (MD: *P* = 0.004, 5%; *λ*_⊥_: *P* = 0.01, 13%) sides. There were no significant differences between the two cohorts in cingulum FA or MTR (Figure 3). 

After adjusting for age and sex, higher EDSS scores and longer disease duration (measured as years since self-reported symptom onset) were associated with decreased fornix FA and increased MD, *λ*_||_, and *λ*_⊥_ (Table 2(a)). Subgroup analysis of the RRMS group showed stronger correlations of longer disease duration with decreased fornix FA and increased fornix MD and *λ*_⊥_ than were found in the MS group as a whole (FA: −0.32, *P* = 0.009, MD: 0.26, *P* = 0.04; *λ*_⊥_: 0.28, *P* = 0.03). Lower fornix FA and MTR, and higher fornix MD and *λ*_⊥_, were correlated with lower PASAT-3 scores, but not with slower 25FTW times. Slower 9-HPT times were also correlated with lower fornix FA and MTR and higher fornix MD, *λ*_||_, and *λ*_⊥_. 

In the averaged cingulum measures, higher EDSS scores were associated with lower FA and higher *λ*_⊥_ (Table 2(b)). Longer disease duration was associated with lower FA and *λ*_||_. Subgroup analysis of disease duration with cingulum measures in the RRMS subgroup was not significant. In the right cingulum, there were no correlations between disability scores and MRI indices. In the left cingulum, however, higher EDSS scores were associated with lower FA and *λ*_||_ and higher *λ*_⊥_. Longer disease duration was associated with lower FA and higher *λ*_⊥_. Lower PASAT-3 scores were associated with lower FA and higher MD and *λ*_⊥_. Slower 9-HPT times were correlated with lower FA and higher MD and *λ*_⊥_. Left cingulum FA, MD, and *λ*_||_ were significantly higher than right cingulum measures. In most cases, correlation coefficients were in the 0.2-0.3 range after adjusting for age and sex.

In order to determine if the relationships observed between fornix MRI indices and PASAT-3 performance were tract-specific, further analysis was performed with corticospinal tract MRI indices. In the corticospinal tract, MRI indices were not significantly associated with PASAT-3 performance after adjusting for age and sex. When corticospinal tract FA was added to the model investigating the relationship between fornix FA and PASAT-3, corticospinal tract FA was not significantly associated with PASAT-3 performance, whereas fornix FA remained significantly correlated with PASAT-3 performance (corticospinal tract FA: *r*: −0.11, *P* = 0.30; fornix FA: *r*: 0.26, *P* = 0.01). The same results were observed in a similar analysis with fornix and corticospinal tract MD, *λ*_⊥_, and MTR. 

The inverse relationship between EDSS and cingulum *λ*_||_ was surprising given the overall increase in *λ*_||_ in the MS cohort relative to healthy volunteers. We found that this relationship could be explained as a phenomenon of age, with different findings in younger and older patients. Specifically, we found that cingulum *λ*_||_ was inversely correlated with age in the whole MS cohort (*r* = −0.23, *P* = 0.005), so that only in the younger MS cases (age < 50th percentile) was left cingulum *λ*_||_ higher than in healthy volunteers (*P* = 0.04). However, in this group there was no association between *λ*_||_ and EDSS. On the other hand, the inverse relationship between *λ*_||_ and EDSS was only observed in older MS cases (age > 50th percentile: *r* = −0.28, *P* = 0.03, *n* = 60); in these cases, *λ*_||_ was on average not significantly abnormal. These results suggest that the mechanisms that lead to increased *λ*_||_ in younger MS cases are not necessarily related to those that are associated with disability in older cases.

## 4. Discussion

Cognitive dysfunction in MS is thought to be associated with gray-matter damage, and MRI studies have provided some evidence supporting this hypothesis [39, 40]. If this is the case, damage to white-matter pathways connecting the gray matter areas responsible for cognition should also be associated with cognitive impairment [41]. In this study, we considered two such pathways: the fornix and the cingulum, both of which connect to the hippocampus. 

Our findings demonstrate that quantitative MRI indices known to be sensitive to the type of tissue damage that occurs in MS, though not specific for particular types of pathology, are abnormal in the fornix and cingulum in MS and correlate with both disease progression and cognitive dysfunction. Specifically, FA and MTR were decreased, and measures of diffusivity increased, in MS cases compared to age-matched healthy controls. In addition, there were correlations between MRI indices and scores on a range of clinical measures, including cognitive tasks such as the PASAT-3. 

Our results provide some support for the notion that tissue damage in the fornix and cingulum is specifically associated with cognitive dysfunction and not an epiphenomenon of overall brain tissue damage. In particular, we found that fornix MRI indices were associated with PASAT-3 scores, whereas corticospinal tract MRI indices were not. Moreover, fornix MRI measures remained predictors of PASAT-3 performance even after corticospinal tract MRI indices were added to the model. Conversely, whereas fornix and cingulum MRI measures were associated with PASAT-3 scores, they were not associated with the 25FTW, which reflects motor disability. 

Consistent with their connections to the hippocampus, the fornix and cingulum play important roles mediating episodic memory and processing speed functions [28]. Deficits in these cognitive domains are commonly observed in MS [5], and our findings may be a result of tissue damage (such as primary axonal pathology, axonal changes resulting from synaptic stripping, and demyelination) in tracts responsible for these functions. Results of a recent analysis of autopsy tissue suggest that hippocampal demyelination may result in decreased in synaptic density and ultimately axon damage [42]. Such damage may be reflected in damage to the fornix, the major hippocampal efferent pathway.

The cingulum findings were asymmetric; only MRI indices in the left cingulum were associated with clinical dysfunction. This result supports previous work in MS demonstrating that the left cingulum is preferentially important for cognitive function and intellectual achievement, including PASAT-3 performance [41, 43, 44]. The reason for this lateralization, however, remains unknown.

Studies in vitro and in animal models have suggested that axon transection can result in congested axons with impaired transport, which is associated with decreased *λ*_||_ [45, 46]. Furthermore, in a longitudinal study following optic neuritis, *λ*_||_ was also shown to decrease in the acute setting and then increase one year after onset [47]. Nevertheless, many studies in MS have demonstrated increases in tract-specific *λ*_||_ despite the presence of widespread axon damage [16, 48], suggesting that decreased *λ*_||_ may only signify axon damage acutely. Consistent with those results, in the full MS cohort studied here, *λ*_||_ in the left cingulum was increased compared to healthy volunteers even after adjusting for the additive effect of age. Paradoxically, however, decreased *λ*_||_ in the same tract was associated with higher degrees of disability. Further investigation showed that these seemingly discrepant findings are related to different results in younger versus older MS cases. Specifically, after stratifying the MS cohort by age, *λ*_||_ was higher only in the younger MS cases; the inverse correlation of *λ*_||_ with disability was restricted to the older MS cases. Since inflammation causes increased overall water diffusivity, one possible explanation for the normalization of *λ*_||_ with age might be reduced inflammation or edema in the older age group. As the confounding effect of inflammation decreases, the sensitivity of *λ*_||_ to ongoing axon damage may increase. 

*Limitations*

A limitation of this study is that the body and pillars of the fornix were used as a surrogate for fornix integrity because only this section of the tract could be reconstructed reliably in scans from all study participants. Despite this, the discrete connectivity of the fornix suggests that DTI indices averaged across the body and pillars of fornix should be representative of the entire tract. Another limitation is the influence of partial volume averaging with CSF due to the small size of the fornix. Increased partial volume averaging in the MS group (due to the inclusion of fewer voxels containing only tract) could accentuate differences from healthy volunteers. In the cingulum, a larger tract than the fornix in which partial volume averaging is less pronounced due to fewer mixed voxels, the presence of lesions could have interfered with tract reconstruction and excluded the most damaged portions of the tracts. Reconstructed tracts were examined visually, however, and tract termination due to lesions was rare. A final and important limitation is that we could not determine cortical lesion volume from the available imaging sequences. Thus, we could not determine whether the observed abnormalities were due to degeneration of tracts connected to gray-matter lesions. In addition, comprehensive cognitive testing was not completed on the full cohort which may allow for elucidation of specific structure function relationships in the different domains of cognition. 

## 5. Conclusions

Our results demonstrate abnormalities within the fornix and cingulum in MS patients which may contribute to cognitive dysfunction in MS. These results further confirm the utility of quantitative MRI to elucidate specific structure-function relationships and to mirror disease progression. Cognitive dysfunction in MS is a common and debilitating symptom of the MS disease process, and our results suggest that damage to white-matter tracts may contribute to this cognitive impairment. This study highlights the importance of axonal connectivity for cognitive processes and suggests that impaired memory processing may impact other brain areas such as the mammillary bodies and septal nuclei via fornix connectivity. Longitudinal studies will be needed to determine the utility of quantitative MRI for predicting cognitive impairment. In addition, quantitative MRI measures may aid monitoring of cognitive impairment in MS patients when used in conjunction with neuropsychological testing.

## Figures and Tables

**Table 1:** Demographics.

	All MS	RRMS	SPMS	PPMS	Healthy controls
No. of participants	101	64	24	13	16
Age, year, mean ± SD	44 ± 12	39 ± 11	55 ± 8	56 ± 7	40 ± 9
Women, *n* (%)	75 (74%)	41 (64%)	17 (71%)	6 (46%)	11 (69%)
Disease duration, years, mean ± SD	11.5 ± 10.2	7.1 ± 6.3	22.6 ± 9.6	12.5 ± 11.2	
Median EDSS (range)	3 (0–8)	2 (0–6.5)	6 (2.5–8)	6 (2.5–8)	
25FTW, sec, median (interquartile range)	4.7 (4.2–35.0)	4.5 (3.9–12.4)	6.5 (5.9–16.5)	7.6 (5.4–35.0)	
9-HPT, sec, median (interquartile range)	24 (20–28)	21 (19–25)	28 (23–35)	32 (26–39)	
PASAT-3, median score (interquartile range)	51 (38–60)	51 (40–60)	49 (39–60)	50 (32–60)	

RRMS: relapsing-remitting multiple sclerosis, SPMS: secondary progressive multiple sclerosis, PPMS: primary progressive multiple sclerosis, EDSS: expanded disability status scale, 25FTW: Timed 25-Foot Walk, 9-HPT: 9-Hole Peg Test, PASAT-3: Paced Auditory Serial Addition Task-3, second version. Disease duration is measured in years since the first reported symptoms attributable to MS.

**Table:** (a)

Outcome variable	FA	MD	Parallel diff.	Perp. diff.	MTR
Disease duration	**−0.22 (0.03)**	**0.26 (0.008)**	**0.25 (0.01)**	**0.26 (0.01)**	−0.17 (0.10)
EDSS	**−0.30 (0.003)**	**0.29 (0.003)**	**0.26 (0.01)**	**0.3 (0.003)**	−0.18 (0.07)
*z*-score 25FTW	0.20 (0.06)	−0.17 (0.11)	−0.13 (0.25)	−0.20 (0.06)	0.008 (0.94)
*z*-score 9-HPT	**0.33 (0.002)**	**−0.35 (0.001)**	**−0.30 (0.005)**	**−0.34 (0.001)**	**0.21 (0.05)**
*z*-score PASAT-3	** 0.24 (0.03)**	**−0.23 (0.04)**	−0.13 (0.24)	**−0.26 (0.02)**	**0.24 (0.03)**

**Table:** (b)

Outcome variable	FA	MD	Parallel diff.	Perp. diff.	MTR
Average cingula					
Disease duration	**−0.22 (0.03)**	0.11 (0.28)	−0.14 (0.18)	**0.19 (0.05)**	−0.05 (0.60)
EDSS	**−0.20 (0.05)**	0.03 (0.78)	**−0.25 (0.01)**	0.17 (0.09)	−0.02 (0.85)
*z*-score 25-FTW	0.04 (0.71)	0.16 (0.12)	0.17 (0.10)	0.05 (0.63)	0.03 (0.78)
*z*-score 9-HPT	**0.22 (0.03)**	**−0.23 (0.02)**	0.06 (0.55)	**−0.27 (0.007)**	0.03 (0.75)
*z*-score PASAT-3	0.18 (0.08)	−0.14 (0.17)	0.11 (0.28)	−0.17 (0.10)	0.07 (0.48)
Left Cingulum					
Disease duration	**−0.27 (0.008)**	0.17 (0.09)	−0.14 (0.18)	**0.23 (0.02)**	−0.09 (0.37)
EDSS	**−0.25 (0.01)**	0.04 (0.66)	**−0.27 (0.006)**	**0.22 (0.03)**	−0.09 (0.40)
*z*-score 25-FTW	0.07 (0.51)	0.09 (0.39)	0.13 (0.21)	−0.02 (0.86)	0.07 (0.47)
*z*-score 9-HPT	**0.28 (0.005)**	**−0.21 (0.03)**	0.10 (0.34)	**−0.28 (0.005)**	0.08 (0.42)
*z*-score PASAT-3	**0.31 (0.002)**	**−0.22 (0.04)**	0.06 (0.54)	**−0.29 (0.004)**	0.15 (0.15)
Right cingulum					
Disease duration	−0.13 (0.19)	0.13 (0.19)	−0.07 (0.50)	0.14 (0.17)	−0.07 (0.47)
EDSS	−0.07 (0.49)	0.03 (0.75)	−0.04 (0.72)	0.06 (0.53)	0.04 (0.68)
*z*-score 25FTW	0.05 (0.60)	0.15 (0.15)	0.18 (0.08)	0.06 (0.58)	0.08 (0.46)
*z*-score 9-HPT	0.11 (0.28)	−0.20 (0.05)	−0.07 (0.52)	−0.17 (0.08)	−0.06 (0.54)
*z*-score PASAT-3	0.09 (0.38)	−0.08 (0.40)	0.05 (0.60)	−0.12 (0.22)	−0.005 (0.96)

FA: fractional anisotropy, MD: mean diffusivity, *λ*_∣∣_: parallel diffusivity, *λ*_⊥_: perpendicular diffusivity, MTR: magnetization transfer ratio, MSFC: multiple sclerosis functional composite, EDSS: Expanded Disability Status Scale, 25FTW: Timed 25-Foot Walk, 9-HPT: 9-Hole Peg Test, PASAT-3: Paced Auditory serial Addition Task-3, second version. Partial correlation coefficients were adjusted for additive effects of age and sex. Significance levels are shown in parenthesis. Results with *P* ≤ 0.05 are shown in boldface.
